# Increased T Regulatory Cells Are Associated with Adverse Clinical Features and Predict Progression in Multiple Myeloma

**DOI:** 10.1371/journal.pone.0047077

**Published:** 2012-10-10

**Authors:** Karthick Raja Muthu Raja, Lucie Rihova, Lenka Zahradova, Maria Klincova, Miroslav Penka, Roman Hajek

**Affiliations:** 1 Babak Myeloma Group, Department of Pathological Physiology, Faculty of Medicine, Masaryk University, Brno, Czech Republic; 2 Department of Experimental Biology, Faculty of Science, Masaryk University, Brno, Czech Republic; 3 Department of Clinical Hematology, University Hospital, Brno, Czech Republic; 4 Hemato-oncology, Department of Internal Medicine, University Hospital, Brno, Czech Republic; Pavillon Kirmisson, France

## Abstract

**Background:**

Regulatory T (Treg) cells play an important role in the maintenance of immune system homeostasis. Multiple myeloma (MM) is a plasma cell disorder frequently associated with impaired immune cell numbers and functions.

**Methods:**

We analyzed Treg cells in peripheral blood (n = 207) and bone marrow (n = 202) of pre-malignant and malignant MM patients using flow cytometry. Treg cells and their subsets from MM patients and healthy volunteers were functionally evaluated for their suppressive property. A cohort of 25 patients was analyzed for lymphocytes, CD4 T cells and Treg cells before and after treatment with cyclophosphamide, thalidomide plus dexamethasone (CTD).

**Results:**

We found elevated frequencies of Treg cells in newly diagnosed (P<0.01) and relapsed MM patients (P<0.0001) compared to healthy volunteers. Also, Treg subsets including naïve (P = 0.015) and activated (P = 0.036) Treg cells were significantly increased in MM patients compared to healthy volunteers. Functional studies showed that Treg cells and their subsets from both MM and healthy volunteers were similar in their inhibitory function. Significantly increased frequencies of Treg cells were found in MM patients with adverse clinical features such as hypercalcemia (>10 mg/dL), decreased normal plasma cell (≤5%) count and IgA myeloma subtype. We also showed that MM patients with ≥5% of Treg cells had inferior time to progression (TTP) (13 months vs. median not reached; P = 0.013). Furthermore, we demonstrated the prognostic value of Treg cells in prediction of TTP by Cox regression analysis (P = 0.045). CTD treatment significantly reduced frequencies of CD4 T cells (P = 0.001) and Treg cells (P = 0.018) but not Treg cells/CD4 T cells ratio compared to pre-treatment.

**Conclusions:**

Our study showed immune deregulation in MM patients which is evidenced by elevated level of functionally active Treg cells and patients with increased Treg cells have higher risk of progression.

## Introduction

Multiple myeloma (MM) is a malignant plasma cell (PC) disorder and is preceded by a pre-malignant stage known as monoclonal gammopathy of undetermined significance (MGUS). MGUS is characterized by lower plasma cell (PC) infiltration (<10%) in bone marrow (BM), <30 g/L of monoclonal protein (M-protein) and absence of organ damage. In contrast, MM patients are characterized by higher PC infiltration (≥10%), ≥30 g/L of M-protein, bone lesions and presence of organ damage [1], [2]. Immune dysfunction is an important feature of MM leading to infections, enhancement of tumor growth and resistance to chemotherapy. Also, decreased level of immune cells (B, CD4^+^ and CD8^+^ cells) has been documented and associated with poor survival of MM patients [3], [4]. In 2003, a study confirmed expansion of regulatory T cells (Treg cells) in cancer patients [5]. Following this study, several studies have shown that Treg cells were associated to impaired immune functions in solid tumors and hematological malignancies [6], [7]. In Treg cells, FoxP3 is considered as a master regulatory molecule [8]. FoxP3 molecule is expressed in thymus-derived Treg cells (natural Treg cells) and peripherally induced Treg cells (CD4^+^CD25hi^+^FoxP3^+^) but not in other induced Treg cells including Tr1 cells and T_H_3 Treg cells [9], [10]. Treg cells suppress other immune cells through contact- dependent and independent mechanisms. Several molecules have been reported for contact-dependent inhibition mechanism, such as CTLA-4 (inhibits antigen presenting cells), lymphocyte activation gene 3 (induces inhibitory signal through MHC II molecules), granzymes (mediate lysis of conventional T cells) and CD95-CD95L (induces apoptosis in conventional T cells) [11], [12], [13], [14]. In contact-independent mechanism, molecules such as IL-10 (attenuates dendritic cells, converts conventional T cells into Tr1 cells), TGF-β and latency-associated peptide (induce FoxP3 expression in conventional T cells), galectin 1 (arrests cell cycle and induces apoptosis in conventional T cells) and CD25 (adsorbs IL-2) plays significant role in suppression [15], [16], [17], [18], [19], [20]. Several studies documented peripheral blood (PB) expansion of functionally active Treg cells in MM [21], [22], [23]. For the first time, our study shows prognostic value of Treg cells in prediction of time to progression (TTP) in MM and their association with adverse clinical features. In addition, this study also acknowledged the findings of previous studies in a larger cohort of patients.

In this study, we quantified and functionally evaluated Treg cells and their subsets from pre-malignant and malignant MM patients. Additionally, we determined the association between MM clinical features and Treg cells. We studied the influence of cyclophosphamide, thalidomide plus dexamethasone (CTD) treatment on the frequencies of Treg cells. Prognostic impact of Treg cells on MM disease progression was also analyzed.

## Methods

### Patients

In this study, patients were included after signing the informed consent form according to the Helsinki protocol, and the study was approved by University Hospital Brno institutional review board. A total of 207 pre-malignant and malignant MM patients were recruited for this study. Based on the diagnosis, patients were classified into: monoclonal gammopathy of undetermined significance [MGUS-22% (45/207)], smoldering multiple myeloma [SMM-3% (6/207)], newly diagnosed multiple myeloma [MM-38% (79/207)], relapsed MM-31% (64/207) and patients in remission-6% (13/207)]. PB samples were collected from all patients but BM samples were collected only from 202 patients due to unavailability of 5 BM samples. PB samples were also collected from 40 healthy volunteers (HVs) as controls. Patients’ characteristics are summarized in Table 1.

**Table 1:** Patients’ characteristics.

	n = 207
Age years; median (range)	66 (33–88)
Male/Female; n (%)	118 (57)/89 (43)
**International staging system; n (%)**	
ISS-1	102 (49)
ISS-2	62 (30)
ISS-3	34 (17)
Unavailable	9 (4)
**Myeloma subtype; n (%)**	
IgG	122 (59)
IgA	40 (19)
IgM	7 (4)
B-J protein	3 (1)
Others	24 (12)
Unavailable	11 (5)
β2-microglobulin mg/L; median (range)	2.81 (0.16–38.2)
Albumin g/dL; median (range)	4.0 (2.11–5.5)
Creatinine mg/dL; median (range)	0.99 (0.53–15.45)
Calcium mg/dL; median (range)	9.28 (6.96–12.8)
Hemoglobin g/dL; median (range)	11.75 (6.4–17.4)
C-reactive protein mg/L; median (range)	4.95 (0–187.1)
LDH µkat/L; median (range)	3.37 (1.57–30.89)
Serum M-protein g/L; median (range)	21.55 (0–88.5)
%of BMPCs; median (range)	2.3 (0–85.5)
% of Normal PCs; median (range)	2.3 (0–94)

Footnote: ISS, International staging system; B-J protein, Bence-Jones protein; LDH, lactate dehydrogenase; M protein, monoclonal protein; and BMPCs, bone marrow plasma cells.

A cohort of 25 newly diagnosed MM patients was analyzed for PB total lymphocytes, CD4 T cells and Treg cells before and after treatment by CTD. Of these 25 patients, 10 patients were treated by autologous stem cell transplantation (SCT) after 4 cycles of CTD induction.

**Figure 1:**
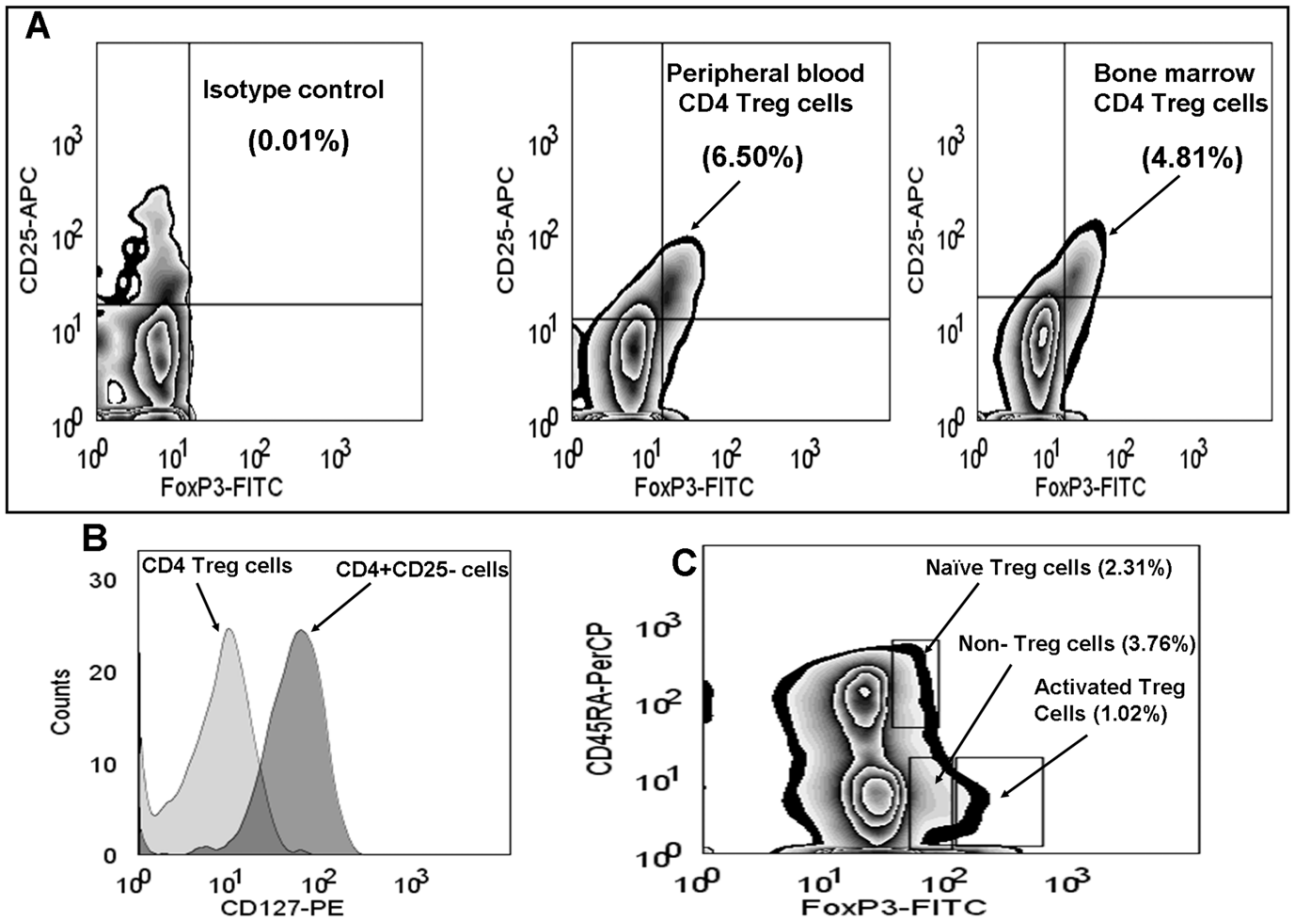
Phenotypic feature of T regulatory cells in peripheral blood and bone marrow. (A) Illustrates isotype-matched control for FoxP3 expression and phenotype of Treg cells (CD4^+^CD25hi^+^FoxP3^+^) from peripheral blood and bone marrow of a MM patient. (B) Histogram shows negative/dim expression for CD127 by Treg cells compared to CD4^+^CD25^−^ cells. (C) Phenotype of naïve (CD4^+^CD45RA^+^FoxP3dim^+^), activated (CD4^+^CD45RA^+^FoxP3hi^+^) and non-Treg cells (CD4^+^CD45RA^-^FoxP3dim^+^) from peripheral blood of a MM patient.

### Sample Preparation

Collected PB and BM samples were processed and used for analysis within 24 hours. Both PB and BM were lysed to remove erythrocytes using BD lysing solution (BD Biosciences, San Jose, California) according to manufacturer’s instructions.

**Figure 2:**
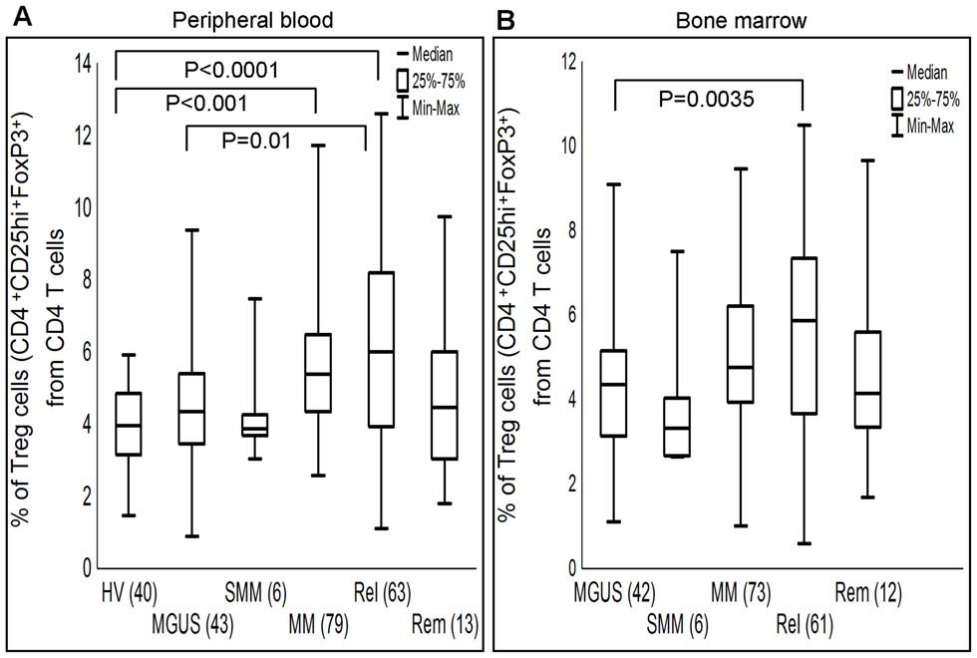
Frequencies of peripheral blood and bone marrow T regulatory cells from pre-malignant and malignant myeloma patients. Peripheral blood (A) and bone marrow (B) Treg cells from different patient cohorts are analyzed and evaluated statistically by Mann-Whitney U test (P≤0.05). Number of samples analyzed in each cohort is given in parentheses. Median, 25^th^–75^th^ percentile and range of data are indicated as horizontal line, box and whiskers, respectively. HVs, healthy volunteers; MGUS, monoclonal gammopathy of undetermined significance; SMM, smoldering multiple myeloma; MM, newly diagnosed multiple myeloma; Rel, relapsed multiple myeloma; and Rem, patients in remission.

### Fluorescence-activated Cell Sorter (FACS) Analysis and Monoclonal Antibodies

After lysis of erythrocytes, 1×10^6^ cells were labeled with the following fluorochrome conjugated monoclonal antibodies: phycoerythrin-cyanin (PE-Cy7)-CD4 (clone SK3), allophycocyanin (APC)-CD25 (clone M-A251) plus with/without phycoerythrin (PE)-CD127 (clone HIL-7R-M21) and (PerCP)-CD45RA (clone MEM-56), and incubated at 4°C for 20–30 min (monoclonal antibodies were obtained from BD Biosciences, San Jose, California and EXBIO Prague, Czech Republic). Then, cells were permeabilized according to eBioscience recommendations (eBioscience, San Diego, California). Finally, cells were labeled with FoxP3 antibody (clone PCH101) conjugated with fluorescein isothiocyanate (FITC) from eBioscience and incubated at 4°C for 30–60 minutes. All prepared samples were measured on BD FACSCanto II.

**Table 2:** Association of T regulatory cells with clinical features of multiple myeloma.

Treg cells	Clinical features (n)	median % (range %)of Treg cells	P value
PB Treg cells	*>10 mg/dL of calcium (36)	5.93 (1.55–11.70)	0.004
	≤10 mg/dL of calcium (167)	4.78 (0.93–12.15)	
BM Treg cells	*>10 mg/dL of calcium (36)	5.88 (1.35–9.30)	0.019
	≤10 mg/dL of calcium (162)	4.62 (0.62–10.46)	
BM Treg cells	* ≤5% of normal PCs (121)	5.3 (0.6–10.4)	0.015
	>5% of normal PCs (79)	4.4 (1.0–10.5)	
PB Treg cells	* IgA (40)	5.96 (2.74–12.05)	0.031
	IgG (122)	5.04 (0.89–9.95)	
BM Treg cells	* IgA (39)	5.73 (2.28–10.43)	0.029
	IgG (120)	4.74 (1.00–10.07)	

Footnote: PB, peripheral blood; BM, bone marrow; and adverse clinical features are marked by asterisk.

In order to evaluate the best sample preparation method for screening of FoxP3 expression, we compared erythrocyte lysis and mononuclear cell (MC) isolation (using Ficoll-Paque) methods in 5 MM patients and 5 HVs. Followed by erythrocyte lysis and isolation of MNCs, cells were labeled similarly as described above (Fig. S1).

### Isolation of T Regulatory Cells and their Subsets

At first, T cell population was isolated from peripheral blood mononuclear cells (PBMCs) using Pan T cell Isolation Kit II (Miltenyi Biotech, Bergisch Gladbach, Germany). Separated T cells were labeled with PE-Cy7-CD4 and APC-CD25. Then, labeled cells were sorted using FACS Aria into two populations: CD4^+^CD25hi^+^ (Treg cells) and CD4^+^CD25^−^ cells (responder cells). To isolate Treg cell subsets, purified T cell population were labeled with PE-Cy7-CD4, APC-CD25 and PerCP-CD45RA. Then, these labeled cells were sorted into four different subpopulations as described previously [24], [25]: CD4^+^CD25^+^CD45RA^+^, CD4^+^CD25hi^+^CD45RA^−^, CD4^+^CD25^+^CD45RA^−^ and CD4^+^CD25^−^CD45RA^+^ (naïve CD4 T cells, responder cells). Purity of sorted cells was >96% for all samples.

**Figure 3:**
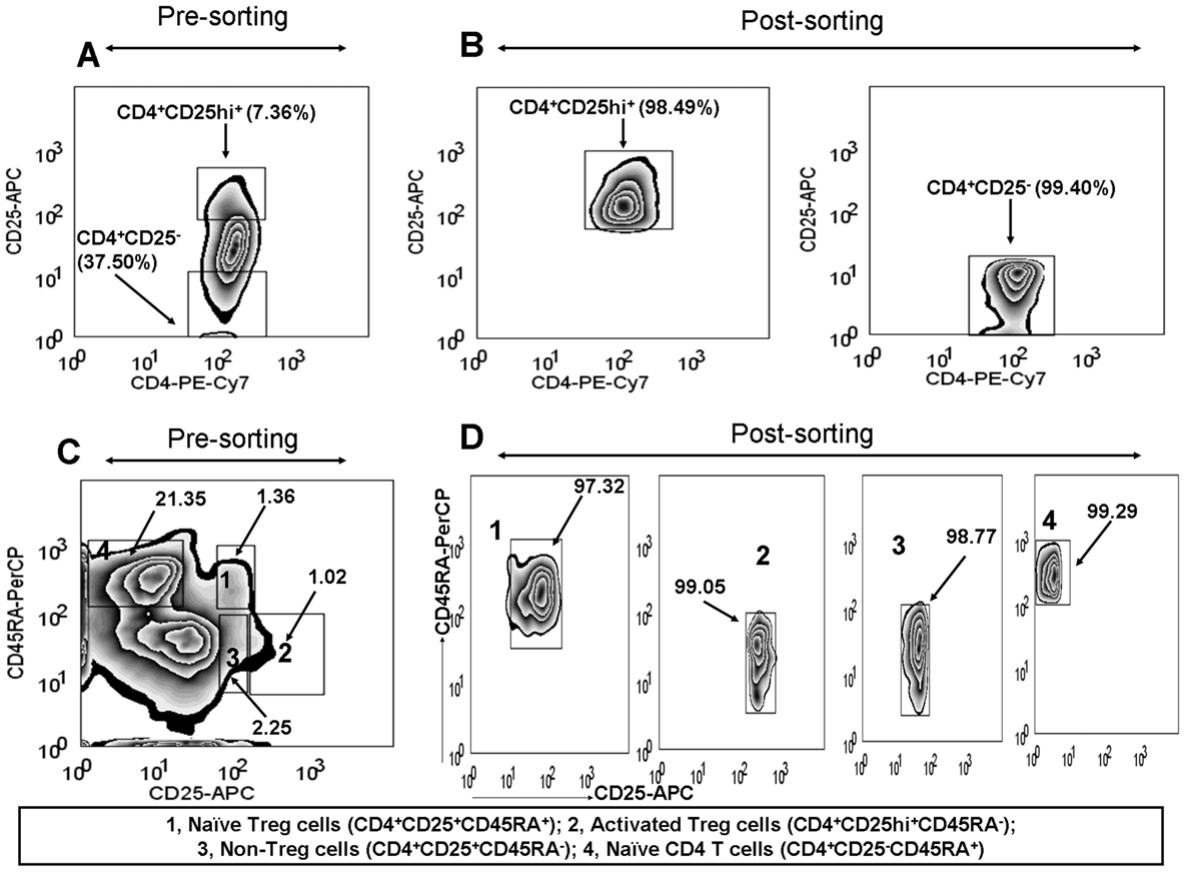
Isolation of T regulatory cells and their subsets. Isolated T lymphocytes were labeled with fluorescent conjugated monoclonal antibodies targeting CD4 and CD25 and sorted by FACS Aria into CD4^+^CD25hi^+^ (Treg cells) and CD4^+^CD25^−^ (conventional T cells). Pre- (A) and post-sorted (B) CD4^+^CD25hi^+^ cells and CD4^+^CD25^−^ cells from a MM patient is shown and purity of sorted cells is represented in percentage. Similarly, to isolate Treg cell subsets, cells were fluorescently labeled for specific antigens (CD4, CD45RA and CD25) and sorted using FACS Aria into four populations: CD4^+^CD25^+^CD45RA^+^ (naïve Treg cells), CD4^+^CD25hi^+^CD45RA^−^ (activated Treg cells), CD4^+^CD25^+^CD45RA^−^ (non-Treg cells) and CD4^+^CD25^−^CD45RA^+^ (naïve CD4 T cells). Pre- (C) and post-sorted (D) Treg cell subsets and naïve CD4 T cells from a MM patient is shown and purity of sorted cells is represented in percentage.

**Figure 4:**
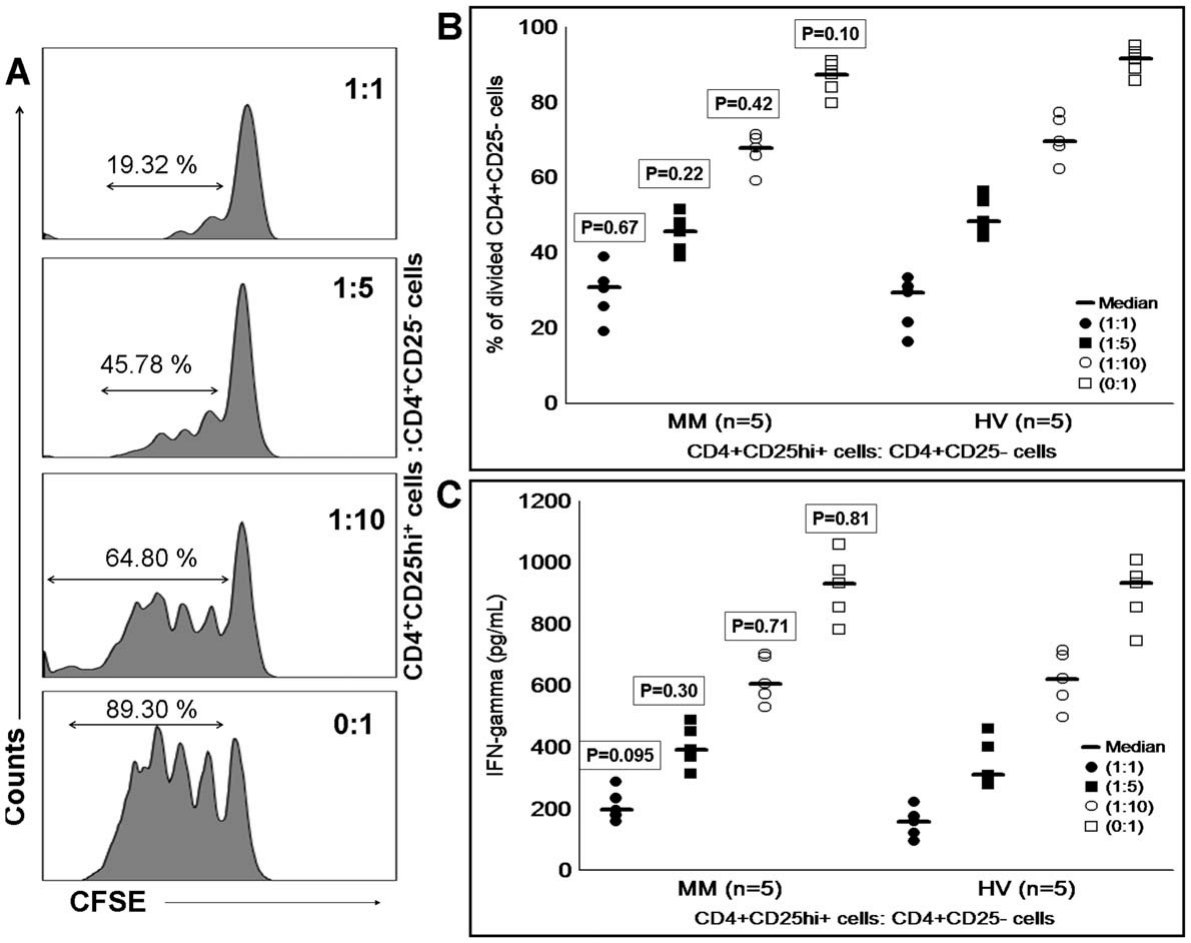
Inhibitory function of T regulatory cells. CFSE labeled CD4^+^CD25^−^ cells were co-cultured with different concentration of Treg cells in the presence of anti-CD3/CD28 beads and accessory cells. After 4 days of co-culturing, (A) based on concentration dependent manner, Treg cells inhibited the proliferation of CFSE labeled CD4^+^CD25^−^ cells which was clearly shown by the dilution of CFSE in FITC channel. In the absence of Treg cells increased proliferation of CFSE labeled CD4^+^CD25^−^ cells was observed. (B) Comparison of MM and HV Treg cells function showed similar level of proliferation inhibition at different concentrations (proliferation/division of CD4^+^CD25^−^ cells is expressed in %). (C) Similarly to proliferation inhibition, IFN-γ concentration from culture supernatant was also decreased based on Treg cell numbers (IFN-γ concentration is expressed in pg/ml). Level of IFN-γ did not differ significantly between MM and HV cohorts from proliferation assays. Mann-Whitney U test was used to assess the statistical difference between MM and HV cohorts. Statistical difference between MM and HV cohorts is indicated by P value. Median is represented by horizontal line, and raw data from each experiment are represented by small dots and squares. CFSE, carboxyfluorescein succinimidyl ester; MM, multiple myeloma; HV, healthy volunteer.

### Assessment of T Regulatory Cells and their Subsets Inhibitory Function

CD4^+^CD25^−^ cells were considered as responder cells and labeled with carboxyfluorescein succinimidyl ester (CFSE). Labeled cells were added at a concentration of 10^5^ cells per well into 24 well round bottom plate. Responder cells were stimulated with anti-CD3/CD28 beads at a ratio of 1∶1 and co-cultured with equal number of autologous accessory cells (preparation of accessory cells: CD3 T cell depleted PBMCs were isolated and treated with mitomycin C as described previously [26]). Finally, different concentrations of CD4^+^CD25hi^+^ cells (0∶1, 1∶1, 1∶5, 1∶10; CD4^+^CD25hi^+^ cells: CD4^+^CD25^−^ cells) were added to responders cells in a final volume of 200–300 µL T cell expansion basal medium containing expansion supplement, 10% of fetal calf serum, 2 mM L-glutamine and 100 U/ml penicillin-streptomycin (obtained from Gibco Invitrogen, Grand Island, New York and Sigma-Aldrich, Germany). Proliferation of responder cells was measured by CFSE dilution on flow cytometer after 4 days of cultivation at 37°C in 5% of CO_2_ atmosphere. To functionally evaluate the subsets of CD4 Treg cells including CD4^+^CD25^+^CD45RA^+^, CD4^+^CD25hi^+^CD45RA^−^ and CD4^+^CD25^+^CD45RA^−^ cells, we performed similar CFSE based proliferation assays as described above. Isolated Treg cell subsets were co-cultured in equal numbers with CFSE labeled naïve CD4 T cells and their proliferation was assessed by CFSE dilution after 4 days of cultivation.

**Figure 5:**
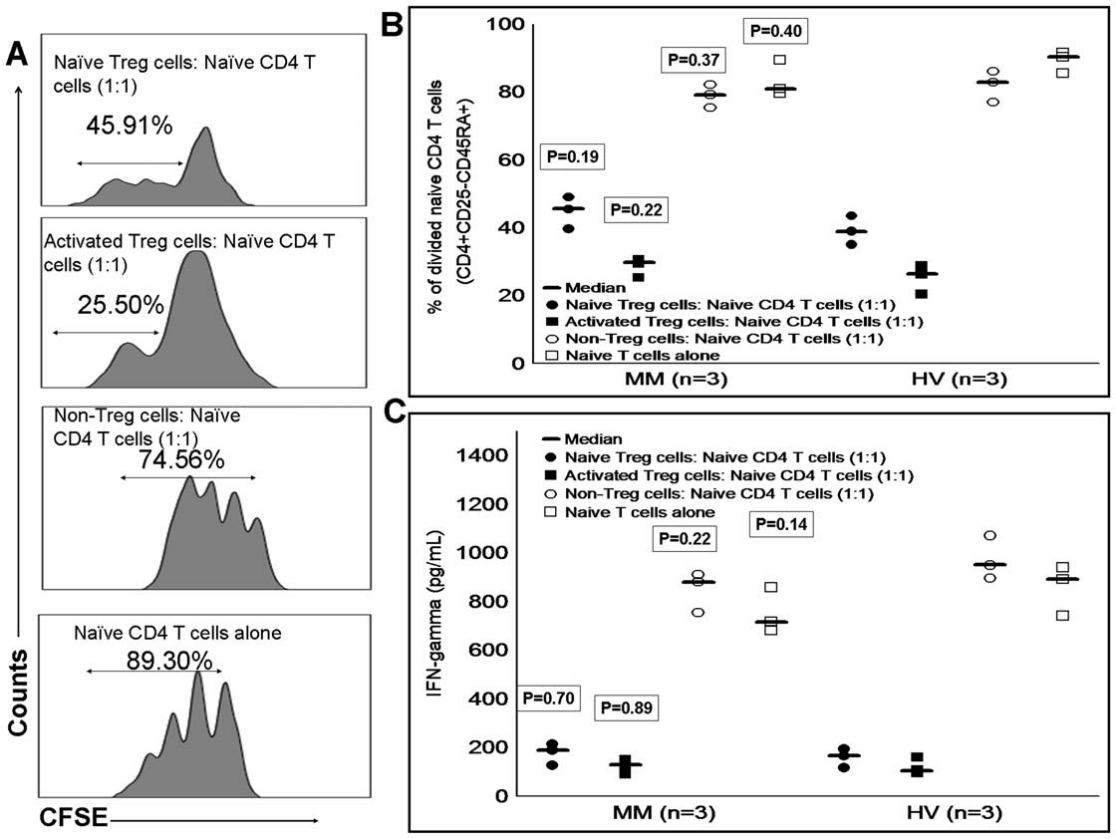
Inhibitory function of naïve and activated T regulatory cells. Similarly, we assessed the suppressive function of naïve, activated and non-Treg cells by CFSE based proliferation assay. Naïve CD4 T cells (CD4^+^CD25^−^CD45RA^+^) were CFSE labeled and co-cultured with naïve, activated and non-Treg cells at a ratio of 1∶1 in the presence of anti-CD3/CD28 beads and accessory cells. (A) Represents proliferation assay from a MM patient. In the presence of naïve and activated Treg cells in the proliferation assay, naïve CD4 T cell proliferation was inhibited but not in their absence/presence of non-Treg cells. This was clearly shown by dilution of CFSE in the FITC channel. (B) In a head to head analysis, proliferation of naïve CD4 T cells between MM patients and HVs did not differ significantly either in the presence or absence of naïve and activated Treg cells in the proliferation assay (proliferation/division of naïve CD4 T cells is expressed in %). (C) Similarly, level of IFN-γ secretion by naïve CD4 T cells did not differ significantly between MM and HV cohorts in the presence or absence of naïve and activated Treg cells (IFN-γ concentration is expressed in pg/ml). Mann-Whitney U test was used to assess the statistical difference between MM and HV cohorts. Statistical difference between MM and HV cohorts is indicated by P value. Median is represented by horizontal line, and raw data from each experiment are represented by small dots and squares. CFSE, carboxyfluorescein succinimidyl ester; MM, multiple myeloma; HV, healthy volunteer.

### Cytokine Profiling

Concentration of IFN-γ from the culture supernatants of proliferation assays were measured using Human IFN-γ Flex set (BD Biosciences, San Jose, California). In brief, supernatants were mixed with IFN-γ capture beads and incubated for 1 hour. This mixture was added with PE detection reagent and incubated for 2 hours. After incubation, the samples were washed and measured on FACS Array.

**Table 3:** Comparison of pre- and post-treatment frequencies of lymphocytes, CD4 T cells and T regulatory cells.

Cell types	Pre-treatment (n = 25)	Post-treatment (n = 25)		P value
	median% (range%)			
Total lymphocytes	16.25 (4.33–41.36)		17.00 (6.58–52.70)	0.44
CD4 T cells	35.45 (23.00–81.33)		27.13 (2.58–54.97)	0.001
Treg cells	5.71 (3.05–9.01)		4.04 (1.67–8.63)	0.018
Ratio of Treg cells/CD4 T cells	0.17 (0.04–0.85)		0.15 (0.06–0.38)	0.31

**Table 4:** Comparison of pre- and post-treatment frequencies of lymphocytes, CD4 T cells and T regulatory cells in relation with treatment response.

Patients with ≥ very good partial response (n = 12)				
Cell types	Pre-treatment		Post-treatment	P value
	median% (range%)			
Total lymphocytes	14.70 (4.33–30.70		19.08 (10.60–45.69)	0.10
CD4 T cells	37.44 (23.00–60.17)		28.34 (7.46–54.97)	0.031
Treg cells	5.46 (3.59–9.01)		3.76 (1.67–7.71)	0.040
Ratio of Treg cells/CD4 T cells	0.15 (0.06–0.38)		0.14 (0.04–0.85)	0.99
**Patients with < very good partial response (n = 13)**				
**Cell types**	**Pre-treatment**	**Post-treatment**		**P value**
	**median% (range%)**			
Total lymphocytes	17.14 (8.27–41.36)		15.57 (6.58–52.70)	0.39
CD4 T cells	34.92 (24.81–81.33)		23.53 (2.58–46.64)	0.026
Treg cells	5.71 (3.05–6.83)		4.20 (1.79–8.63)	0.22
Ratio of Treg cells/CD4 T cells	0.16 (0.06–0.27)		0.18 (0.05–0.74)	0.18

### Data Analysis

Treg cells and their subsets were analyzed from CD4^+^ T cells using FACSDIVA software 6.1.2. IFN-γ secretion was analyzed using FCAP Array software.

**Figure 6:**
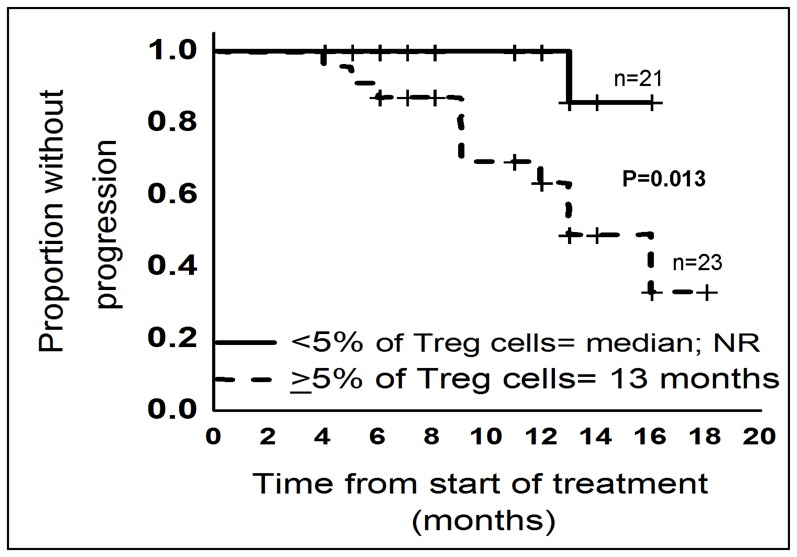
Time to progression analysis according to T regulatory cells. TTP analysis from 44 previously untreated myeloma patients showed patients with ≥5% of Treg cells have shorter time for progression. Kaplan-Meier curves demonstrate TTP according to ≥5% and <5% of peripheral blood Treg cells. TTP, Time to progression; n, number of patients; NR, not reached.

**Table 5:** Univariate Cox regression analysis for factors influencing time to progression in multiple myeloma.

Prognostic factors	Hazard ratiofor TTP	95% CI	P value
Age; ≥65 years	0.8	0.4–2.6	0.53
Serum M-protein; ≥30 g/L	1.4	0.3–6.8	0.65
β2-M; ≥3.5 mg/L	1.1	0.3–3.7	0.87
Albumin; <3.5 g/L	0.8	0.2–2.5	0.64
Creatinine; ≥2 mg/dL	1.2	0.3–5.4	0.84
Calcium; >10 mg/dL	0.3	0.0–2.4	0.26
Hemoglobin; <10 g/dL	0.6	0.2–2.1	0.46
BMPCs; ≥10%	0.7	0.2–2.7	0.61
N-PCs; >5%	2.0	0.4–9.5	0.39
ISS stage	2.4	0.3–18.6	0.41
**PB Treg cells ≥5%**	8.2	1.0–63.8	0.045
BM Treg cells ≥5%	2.5	0.7–9.6	0.37

Footnote: CI, confidence interval; ISS, international staging system; M protein, monoclonal protein; β2-M, β2 microglobulin; BMPCs, bone marrow plasma cells; N-PCs, normal plasma cells.

### Statistical Analysis

Data were expressed in median and range percentages for Treg cells and their subsets. Non-parametric analyses were used including Mann-Whitney U test and Kruskal-Wallis test to evaluate the difference between two or more independent groups. Association between two continuous variables was estimated by Pearson correlation coefficients (r). TTP was calculated from start of treatment to progression; patients who did not progress were censored based on their last visit. Kaplan-Meier method was used to plot survival curves and difference between curves was calculated by Log-rank test. Cox regression analysis was used to evaluate the prognostic effect of Treg cells on TTP. To evaluate the difference between groups, P value of ≤0.05 was considered as significant. All statistical analyses were performed with STATISTICA software (9.0).

## Results

### Phenotypic Analysis of Treg Cells and their Subsets

Treg cell phenotype from HVs, pre-malignant and malignant MM patients was identified as CD4^+^CD25hi^+^FoxP3^+^ (Fig. 1A). In 7 myeloma patients, we also screened CD127 expression on Treg cells and results showed dim/negative expression for CD127, which is one of the features of Treg cells (Fig. 1B). Based on the expression of CD45RA and FoxP3 markers by CD4 T cells, three subsets of Treg cells were identified as described previously [24], [25]: naïve Treg cells (CD4^+^CD45RA^+^FoxP3^dim+^), activated Treg cells (CD4^+^CD45RA^−^FoxP3^hi+^) and non-Treg cells (CD4^+^CD45RA^−^FoxP3^dim+^) (Fig. 1C).

### T Regulatory Cells and their Subsets are Elevated in Multiple Myeloma Patients and Associated with Adverse Clinical Features

Quantification analysis showed that PB Treg cells were significantly elevated in patient cohort compared to HVs [5.22% (0.89%–12.60%) vs. 3.96% (1.47%–5.90%); P<0.001]. Frequencies and statistical results of PB and BM Treg cells of HVs and patient cohort with differential clinical representations are summarized in Fig. 2. An evaluation was done for PB Treg cells by pooling newly diagnosed, relapsed and patients in remission together as a MM cohort; this pooled cohort did not show statistical significance against MGUS and SMM, while it was highly significant against HVs (P<0.001). Unlike PB Treg cells, significant decrease in BM Treg cells was observed in MGUS cohort compared to pooled MM cohort [4.35% (1.10%–9.09%) vs. 5.25% (0.58%–10.50%); P<0.01]. Both PB and BM Treg cells from MGUS cohort were significantly decreased compared to relapsed MM cohort (P = 0.01, P = 0.0035, respectively) (Fig. 2). In correlation analysis, frequencies of PB and BM Treg cells from patient cohort showed strong correlation (r = 0.79; P<0.001).

Next, we analyzed Treg cell subsets including naïve, activated and non-Treg cells from PB of 5 MM patients and 5 HVs to evaluate whether changes occurred in these subsets. Comparative analysis showed that frequencies of naïve and activated Treg cells were significantly increased in MM patients compared to HVs [naïve Treg cells: 1.94% (1.04–2.95) vs. 0.87 (0.55–1.49); P = 0.015, activated Treg cells: 1.02 (0.56–2.59) vs. 0.54 (0.42–1.21); P = 0.036]. But non-Treg cells from MM patients and HVs did not differ significantly [4.15% (2.78–5.86) vs. 3.64 (1.94–5.09); P = 0.19].

Then, we analyzed the association between Treg cells and clinical features of MM. Analysis showed that MM patients with adverse clinical features, such as hypercalcemia (>10 mg/dL of calcium), lower normal PC count (≤5%) and IgA myeloma subtype had significantly increased frequencies of Treg cells (Table 2). Based on international staging system (ISS) of MM, we observed insignificant increase in frequencies of PB and BM Treg cells (data not shown).

Of note: Comparison of MC isolation and erythrocyte lysis methods showed similar mean fluorescence intensity of FoxP3 expression in Treg cells (Fig. S1).

### T Regulatory Cells and their Subsets (Naïve and Activated) are Potential Suppressors of CD4 T Cells

For functional studies of Treg cells and their subsets, highly purified cells (>96%) were used (Fig. 3) We assessed Treg cells (CD4^+^CD25hi^+^) from 5 newly diagnosed MM patients and 5 HVs for their suppressive function against responder CD4 T cells (CD4^+^CD25^−^). Proliferation of CD4^+^CD25^−^ cells was inhibited in the presence of Treg cells, and inhibition was observed in dose-dependent manner (Fig. 4A). Inhibitory activity of Treg cells from both MM and HV cohorts was comparable (P>0.05) (Fig. 4B). Kruskal-Wallis test showed that dose-dependent inhibition of proliferation by Treg cells was statistically significant for MM cohort (P<0.001) and HV cohort (P<0.001). Consistent with the finding of proliferation inhibition by Treg cells, culture supernatant from MM cohort (P<0.01) and HV cohort (P<0.01) also showed decreased concentration of IFN-γ with increased numbers of Treg cells (Fig. 4C).

Due to increase in the number of Treg subsets including naïve and activated Treg cells in MM patients, we intended to perform functional studies to evaluate if these Treg subsets possess suppressive function. Functional data showed that naïve and activated Treg cells from MM and HV cohorts significantly inhibited the proliferation (Fig. 5A) and IFN-γ secretion by naïve CD4 T cells compared to their absence in the proliferation assay (Kruskal-Wallis test; P<0.05). A trend of increased inhibition of proliferation and IFN-γ secretion was observed in activated Treg cells from MM and HV cohorts compared to naïve Treg cells. In comparative analysis, proliferation and IFN-γ secretion by naïve CD4 T cells did not differ significantly between MM (n = 3) and HVs (n = 3) either in the presence/absence of naïve and activated Treg cells (Fig. 5B, 5C). On contrary to naïve and activated Treg cells, co-culturing of non-Treg cells with naïve CD4 T cells showed that proliferation and IFN-γ secretion by naïve CD4 T were similar compared to their absence. Taking these data together clearly suggest that Treg cells and their subsets (naïve and activated Treg cells) are functional in MM patients as similar to HVs.

### Influence of Cyclophosamide, Thalidomide Plus Dexamethasone Treatment on T Regulatory Cells

Post-CTD treatment assessment showed significant reduction in the frequencies of CD4 T cells and Treg cells compared to pre-treatment, whereas lymphocyte frequencies and ratio of Treg cells/CD4 T cells were similar (Table 3). With regard to treatment responses, ≥very good partial response (VGPR) was achieved in 12 patients (complete response-3/12 plus VGPR-9/12) and 13 patients achieved <VGPR (partial response-7/13, minimal response-1/13 and progressive disease-5/13). Patients with ≥VGPR showed significant reduction in the frequencies of CD4 T cells (P = 0.031) and Treg cells (P = 0.040) after treatment compared to pre-treatment. However, patients with <VGPR showed significant reduction only for CD4 T cells (P = 0.026) (Table 4). Treg cells/CD4 T cells ratio did not differ significantly after treatment compared to pre-treatment in patients with ≥VGPR and <VGPR.

### Peripheral Blood T Regulatory Cells Predict Time to Progression

TTP data were available only for 44/79 (56%) of newly diagnosed MM. From 44 patients, 88% of patients were treated with CTD, and remaining 12% of patients were treated with different combinations including bortezomib, adriamycin plus dexamethasone, and cyclophosphamide plus dexamethasone. The median follow-up of this cohort was 11 months (range: 4–18 months). A cut-off value of 5% was selected based on median percentage of Treg cells from 44 patients. Log-rank test showed that patients with ≥5% of PB Treg cells had shorter TTP compared to patients with <5% of PB Treg cells (13 months vs. median not reached; P = 0.013) (Fig. 6). In univariate Cox regression model, only PB Treg cells but not other variables (ISS staging, β2M, albumin, calcium, creatinine, BMPCs, normal PCs, hemoglobin, M-protein and BM Treg cells) showed prognostic value of predicting TTP (P = 0.045) (Table 5). Unfortunately, we were unable to perform multivariate Cox regression analysis due to only one variable (PB Treg cells) showed significance in univariate analysis and also no other variables were even closer to significant value of P≤0.1.

## Discussion

Treg cells play an important role in maintaining immunological homeostasis and any imbalances leads to impaired immune functions. It is accepted that Treg cells are expanded in hematological and non-hematological malignancies. Increased FoxP3 expression and Treg cell elevation are generally considered to be poor outcome markers in various cancers, including Hodgkin’s lymphoma, follicular lymphoma, breast cancer, gastric malignancies, and ovarian cancers [27], [28], [29], [30], [31]. For the first time in MM, we were able to demonstrate that increased frequencies of Treg cells (≥5%) predicted shorter TTP. Very recently, a study also showed that high number of Treg cells from MM patients were associated with poor survival [32]. However, several questions are yet to be answered in MM; do tumor cells enhance the expansion of Treg cells during progression or is progression induced by Treg cells due to suppression of anti-tumor responses? We believe that both processes are mutually inclusive because several *in vitro* and animal model studies in other cancers documented that tumor and tumor infiltrating cells derived factors such as CCL2, prostaglandin 2 (PGE2), H-ferritin, indoleamine 2, 3-deoxyginase (IDO) and IL-10 recruit and enhance Treg cells in the tumor microenvironment and thereby, increase the risk of tumor progression [27], [33], [34]. In MM, a study showed that dendritic cells enhanced the expansion of Treg cells *in vitro* and *in vivo* [35]. However, it is still unknown how Treg cells are influenced by tumor cells/tumor microenvironment of MM patients.

In terms of frequencies/numbers of Treg cells, we found that PB Treg cells were expanded in MM, which was consistent with other studies [21], [22], [23]. Moreover, we and others confirmed a paradigm of decrease in Treg cells from MGUS cohort compared to MM cohort [21], [22], [23]. Our report showed significantly elevated levels of Treg cells in relapsed MM patients compared to MGUS which was not previously demonstrated by other studies. Some studies demonstrated that control group had elevated frequency of Treg cells compared to MGUS and MM cohorts [36], [37]. These opposing results may be due to Treg cell identification strategy; Prabhala et al identified Treg cells as CD4^+^FoxP3^+^ [36], Gupta et al characterized Treg cells with the inclusion of CD127 in their gating [37], we and Feyler et al identified Treg cells as CD4^+^CD25hi^+^FoxP3^+^ [23], and Beyer et al identified Treg cells using CD4 and CD25 markers [21]. In SMM, our data showed insignificant decrease in PB and BM Treg cells compared to other cohorts (HVs, MGUS, and MM). However, due to a small number of patients in this cohort, no explanation is conclusive. Our data showed similar level of proliferation inhibition by Treg cells from MM patients compared to HVs, which was corroborated by IFN-γ production. This observation is consistent with other studies [21], [22], [23], [37] but in contrary to Prabhala et al, who documented that Treg cells from MGUS and MM patients have reduced inhibitory function [36]. This difference might be due to the use of CD25 depleted whole PBMCs as responder cells. Similarly to *in vitro* studies, a recent observation showed that in MM after allogenic SCT, donor-derived Treg cells reconstituted in BM were functional and also enhanced the survival of transplant without graft versus host disease [38].

Next, we focused on analysis of Treg cell subsets including, naïve and activated Treg cells in MM patients to understand their expansion and suppressive function. Our data clearly showed that from 5 MM patients, naïve and activated Treg cells were significantly increased compared to HVs which corroborated previous finding [21]. Functional data revealed the ability of these Treg subsets (naïve and activated Treg cells) from MM patients to suppress the proliferation and IFN-γ secretion of naïve CD4 T cells in similar fashion to HVs. Recently, similar proliferation inhibition by Treg cell subsets has been reported in colorectal cancer [39]. Beyer et al TREC (T-cell receptor excision circle) analysis showed that increased numbers of naïve and memory Treg cells in MM were due to peripherally expanded Treg cells rather than thymus derived Treg cells [21]. Our data from MM patients and HVs have shown that CD4 T cells which are FoxP3^dim+^ and CD45 RA^−^ (non-Treg cells) did not possess suppressive function. This observation is consistent with the previous finding [25]. Taking these data together clearly suggest that naïve and activated Treg cells in MM patients are expanded and functionally active.

We intended to screen the association between clinical features of MM and Treg cells. Our data showed significant increase in Treg cells from MM patients with adverse clinical features, such as hypercalcemia, lower normal PC count and IgA myeloma subtype. Hypercalcemia is a common adverse feature in MM associated with bone resorption [40]. Presumably, the possible relation between Treg cells and hypercalcemia could be explained by the cytokine production including tumor necrosis factor (TNF-α), vascular endothelial growth factor (VEGF), colony stimulating factor (CSF), IL-10 and TGF-β at the tumor site which prominently recruit osteoclasts and might also aid in expansion of Treg cells. We suspect that this mechanism might provoke the increase of osteoclasts and results in bone lytic lesions and subsequently hypercalcemia occurs [23], [40], [41]. Patients with IgA myeloma had significant increase in frequencies of Treg cells compared to patients with IgG myeloma. From this finding, we believe that IgA myeloma patients might have stronger immunological suppression than IgG myeloma patients which could also influence survival. To support this hypothesis, several clinical data reported that IgG myeloma patients had better prognosis than IgA myeloma patients but difference between IgG and IgA myeloma patients with regard to immune status/performance and functions are yet to be revealed [42], [43]. There are reports available showing that stage dependent increases of Treg cells in MM and B-CLL and our data also corroborate this finding but lacks statistical significance [23], [44].

In our study, patients treated by CTD showed significant reduction of CD4 T cells and Treg cells compared to pre-treatment. Similar results were observed in B-CLL patients who were treated by fludarabine plus thalidomide [45]. Effects of cyclophosphamide on Treg cells were also observed in animal models [46], [47]. We believe that reduction of CD4 T cells is due to cytoreductive and cytotoxic effects of cyclophosphamide. Apart from cyclophosphamide, patients were also benefited by thalidomide inclusion, which induces anti-tumor and immunomodulatory effects [48]. We analyzed ratio of Treg cells/CD4 T cells to evaluate the CTD treatment effects on Treg cells. Analysis showed that the ratio of Treg cells/CD4 T cells did not change significantly after treatment. This might be due to low effect of thalidomide on Treg cells in comparison to other immunomodulatory drugs (lenalidomide and pomalidomide) which was already shown in an *in vitro* study [49]. These data suggest that Treg cells are reduced due to overall reduction of CD4 T cells. Moreover, patients with ≥VGPR after CTD treatment had significantly reduced Treg cells compared to patients with <VGPR. In contrast to our observation, Gupta et al showed that patients who responded to therapy had increased Treg cells but not in patients with stable and progressive disease [37]. This conflicting result might be due to inclusion of heterogeneously treated patients in their study.

In summary, our study shows that Treg cells and their subsets from PB of MM patients are elevated and functional. Also, our study demonstrates the prognostic value of Treg cells in predicting the progression risk of myeloma patients. Post-CTD treatment result showed reduction of Treg cells and CD4 T cells but Treg cells/CD4 T cells ratio was similar. These findings suggest that tumor cells in MM patients might evade immune surveillance via increasing functionally suppressive Treg cells either in the PB or tumor bed. Taking all these observations into consideration, we propose that treatment targeting Treg cells might be beneficial for MM patients.

## Supporting Information

Figure S1 **Comparison of FoxP3 expression between mononuclear cell isolation method and erythrocyte lysis method.** A total of 10 cases including 5 MM and 5 HVs peripheral blood samples were collected, and cells were prepared for FoxP3 analysis by MC isolation method and erythrocyte lysis method as described in methodology section. (A) and (B) represent density plots of Treg cell analysis from a sample that was prepared by MC isolation method and erythrocyte lysis method, respectively. In all 10 cases, the expression of FoxP3 was highly similar (no significant difference) between MC isolation method (mean± SD of MFI and % of Treg cells- 766.10±163.10 and 5.99±1.25) and lysis method (mean± SD of MFI and % of Treg cells-748.80±148.09 and % of Treg cells-5.91±1.17). Pearsons’ correlation analysis showed very strong significant association (r>90; P<0.0001) between MC isolation method and erythrocyte lysis method for MFI of FoxP3 expression (C) and frequencies of Treg cells (D). MC, mononuclear cells; MFI, mean fluorescence intensity.
(TIF)
