# Supplementary figures and images for: Increased T Regulatory Cells Are Associated with Adverse Clinical Features and Predict Progression in Multiple Myeloma
Source: PLoS One. 2012 Oct 10;7(10):e47077. doi: 10.1371/journal.pone.0047077 (PMC3468567; doi:10.1371/journal.pone.0047077)

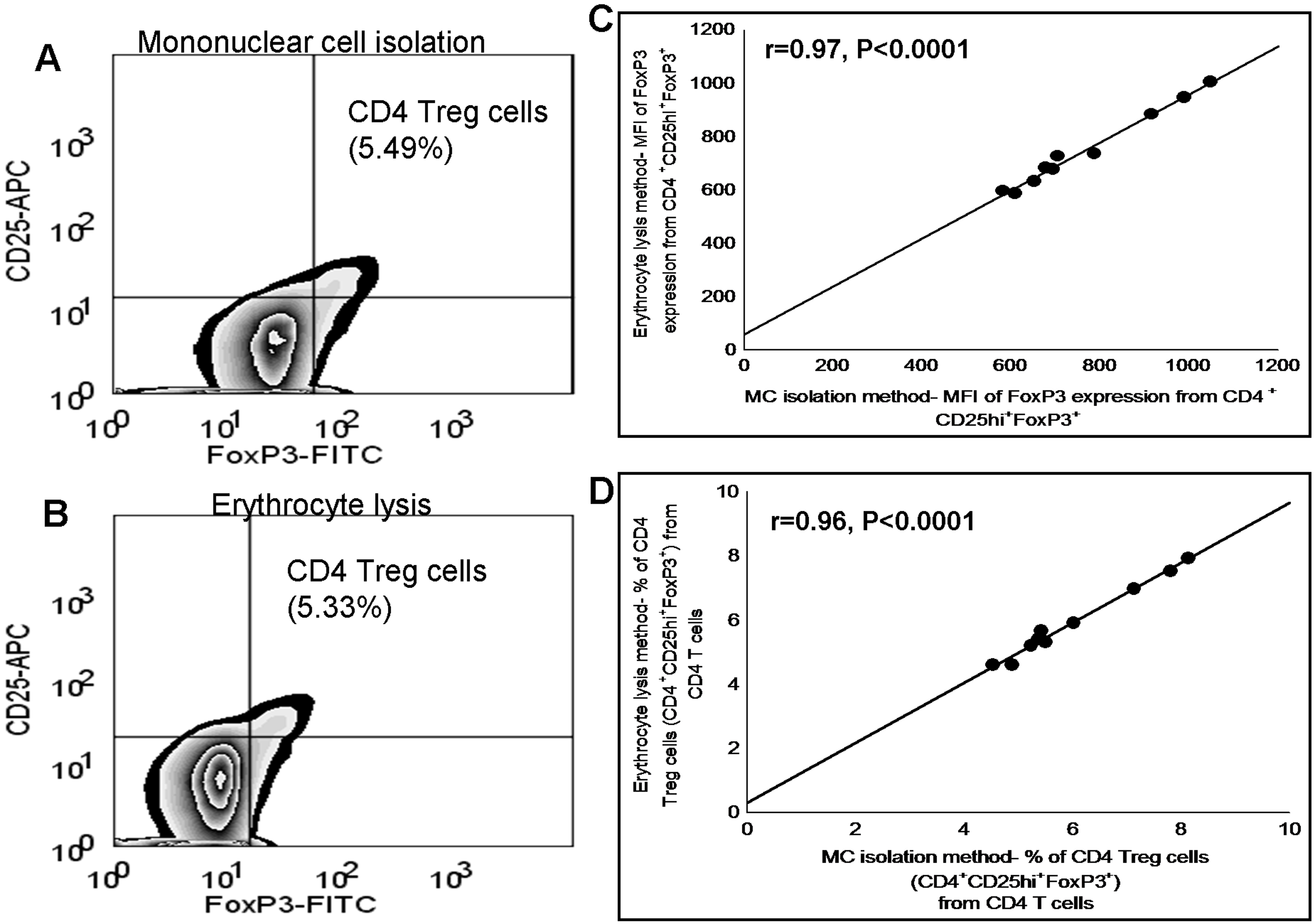

Supplement: Figure S1 — Comparison of FoxP3 expression between mononuclear cell isolation method and erythrocyte lysis method. A total of 10 cases including 5 MM and 5 HVs peripheral blood samples were collected, and cells were prepared for FoxP3 analysis by MC isolation method and erythrocyte lysis method as described in methodology section. (A) and (B) represent density plots of Treg cell analysis from a sample that was prepared by MC isolation method and erythrocyte lysis method, respectively. In all 10 cases, the expression of FoxP3 was highly similar (no significant difference) between MC isolation method (mean± SD of MFI and % of Treg cells- 766.10±163.10 and 5.99±1.25) and lysis method (mean± SD of MFI and % of Treg cells-748.80±148.09 and % of Treg cells-5.91±1.17). Pearsons’ correlation analysis showed very strong significant association (r>90; P<0.0001) between MC isolation method and erythrocyte lysis method for MFI of FoxP3 expression (C) and frequencies of Treg cells (D). MC, mononuclear cells; MFI, mean fluorescence intensity. (TIF) [file pone.0047077.s001.tif]
